# Satan in Society

**Published:** 1871-02

**Authors:** Volta Reeke


ARTICLE VIII. — *Satan in Society.* By a Physician. (First American Edition, and we hope the last.) By VOLTA REEKE.

It is with some pain, and loathing too, that we finish a hasty perusal of this work, handed us a few days ago by a friend. Pain, because we have much faith in the inborn purity and goodness of human nature; loathing, that a man should exist, and he a physician, to produce such a mixture of falsehood, illogical trash, and bawdy nonsense, and then have the assurance to declare that he hopes and desires it may be read by all classes of the community—male and female, young and old—as an almost specific cure for the evil ways they are pursuing, and for the vices they have acquired. It is on a par with several books of like character, written in the last few years, under the cover of scientific authority, insidious, untruthful, with clap-trap titles, and made for sale. It is not one whit more decent than the *Police Gazette*, and other low pictorials of the phosphorescent style, and stealing into the family circle under such disguise, does more to corrupt and degrade, and turn the thoughts of the growing generation into impure channels, than all other causes or associations put together. He quotes the misogamists, the misanthropists, the *debauchees*—Balzac, Michelêt, Tissot, Legouvé, and others—of the French school, to sustain him in the many peculiar opinions advanced; yet despite such philosophical aids—and many of them not used in their proper connections—we cannot see one position legitimately tenable, not one argument spun to a perfectly logical conclusion, as his premises are false to build on, are opposed to the observation and experience of many learned men, and hence, end in the *reductio ad absurdum.*

We speak particularly here of the first six chapters; the remainder of the work is compiled from the best authorities, well written, and unfortunately gives an air of respectability to the whole. There pervades, throughout, a vein of christian sentiment, which is one-half bigotry and one-half cant, throwing a very strong doubt over the author's sincerity, and leading one irresistibly to the conclusion, that money is the sole object, and not the welfare of society.

It is unquestionably an evidence of caution, and sense too, that our author resisted so manfully the solicitations of his many admirers and friends to publish his name in connection; for we agree with him that, if the public has any self-respect or decency left, it might render a verdict which would seriously interfere with the harmonious relations of his extensive *clientèlle*, and endanger, to the extent of many dollars, his future welfare and happiness.

The first point that struck our attention was his quotation from Legouvé, and on the next page from Balzac, where they discuss the wedding night, and all assume that the precipitate consummation of marriage is a *rape*, and that instead of love and reverence following, only hatred and disgust is engendered. To sustain such a position for a moment, we must of course pre-suppose every woman a timid, ignorant fool, and every man a brute of the worst stripe, who rushes to the attack like an aggressive stallion, utterly regardless of the feelings of her, whom in love and honor, he has just taken for weal or woe, and without any of those marital endearments necessary to soothe, and gain consent. This is to take entirely a too brutal view of the question, and is not true in fact. Of the various causes producing estrangement among married people in after life, this is assuredly the least frequent. And the "skeleton in the closet" does not wag his grinning jaws, nor shake his clanking bones, because the husband worships prematurely or too often at the shrine of Venus, but rather the converse holds good. Legouvé and Balzac, are very Frenchy, and speak more from a false morbid philosophy, than from the actual experiences of life, and when the latter lays it down as an axiom that, "the husband who begins with his wife by a *rape* is a lost man: he will never be loved;" and our author groans out plaintively: "Alas! what else is the ordinary consummation of marriage, but a legalized rape?" we can only exclaim, Shade of Priapus! howl out your dissent to these madmen and pretenders, and tell them that rapes are uncommon and unfashionable now-a-days, and never occur except when extortion or blackmailing is the object. Approach, and ask in fitting terms any married woman—and we mean woman in its truest and most acceptable sense—she will laugh at such an absurdity; and will tell you, moreover, that such precipitation is not only expected, but allowable, and that she who expects otherwise is a rare exception. We know that brutes exist, capable of committing almost any atrocity, and likewise capable of a *rape* on their own or others' wives, but they are not the general rule, as our author holds, being few and far between. And in connection with this subject, let us advert to his remarks on the nuptial chamber, in his admonition to husbands. Here again, his advice to that individual upon crossing the sacred threshold, gives one the impression that his experience—and to read his cases, it is really the fact—of married men proves them all lecherous satyrs, who no sooner know their brides abed, than with a whisk of their tails (coat) they enter, barricade the doors, hardly stop to take off their breeches, and furiously attacking their shrinking victims—who of course are not aware that *men* differ physically—soon turn the fair nuptial chamber into a slaughter-pen, tearing, bruising, mutilating without mercy, and only ceasing through exhaustion or satiety. We have very little to reply to such nonsense, except to assure our author that most married men find room enough to prevent all this mutilation, and all recognize the fact that the tissues to be forced are very elastic, and will stretch a yard before they will tear an inch. Should much soreness be left, a dash or two of cold water, and several ounces of Goulard's Extract will complete the job. He speaks in fitting terms of those "diabolical inventions," and of every artificial way of preventing conception, but invalidates his whole argument, and casts doubt upon his sincerity, when, a few pages further on, he tells all who do not wish children, of fourteen days grace between the menstrual flows, when they are exempt from danger, and when they can peg away like guinea-pigs, *ad nauseam;* at the same time he exclaims, if this interval is not sufficient, "*where the necessity exists*, the man is a beast and should be resisted at all hazards." Before discussing this further, we will introduce an anecdote to illustrate his position: Two planters were returning by the river to their respective plantations, the one accompanied by a fine looking, athletic body servant, whom the other noticing, requested to be left on his place for a few weeks, as he much desired some of his stock. The owner being agreeable, called up Jack, and submitted the proposition, to which Jack replied, scatching his head reflectively: "Well now you see, Massa, I'se willin', den dey don't call upon me more dan eight, ten, or twelve times in de night; but, if dey wants me more dan dat, an' wants me to make a *beast* of myself, I can't do it, no how." Jack was right, and would not be made a beast of, and our author is right in counseling resistance "at all hazards" to the husband who would make a beast of himself. But, to digress no further, we shall view this question in a still more ultra light. Firstly, that the conjunction of the sexes is a wise ordinance of the Almighty, to perpetuate the human race, and the mere physical pleasure is subordinate, and in no way to be taken into consideration. Secondly, that any measure, artificial or otherwise, (whether by "diabolical inventions," or by advantage of the fourteen days season) to prevent conception, is both sinful and criminal; and, Thirdly, if children are desired, go ahead and beget them—herein lies no sin—but if otherwise, there must be absolute and unconditional continence, and if this can be secured in no other way, castration must be resorted to.

We think our argument is far the soundest, and not only allows of no deviation from the straight and narrow path, which every christian should follow, but brings itself to a strict logical conclusion. Though no churchman, which our author evidently claims to be, we do not hesitate to urge him to adopt our view in his next edition, as it is fast becoming necessary to regenerate the world in this respect, and that the conjunction of the sexes, without a proper natural compensation, must be "resisted at all hazards."

As to abortions, miscarriages, etc., no one could look upon them with more horror than ourselves, but when the author, taking the ultra church doctrine, declares that they are never permissible, we know him to be insincere, or a bigot. Because a man's judgment may be faulty, and mistakes sometimes made, must no interference take place, and mother and child both be sacrificed? My good sir, you know that there is no better established axiom in midwifery, than "that the child should be sacrificed to the mother," an axiom established by the observation, experience, and humanity of the most learned men many years back, and endorsed by the good sense of every one, who takes a proper view of the comparative worth of both lives; and it does not alter this fact a particle, because incompetent men creep into the profession, and sacrifice lives, that could be saved by the better educated; it simply warns us of the great responsibility placed upon our shoulders, and that it is our duty to study, and reflect, and counsel, to fill conscientiously the vocation to which we have been called. This reasoning is similar to the withholding of anæsthetics from lying-in women, simply because God had declared they should bear children in great pain; and might be equally applied to the practice of every medical man, since illness descends to us "as the sins of our fathers," or comes to us from the obvious infraction of moral or physical laws.

The author dwells learnedly upon the education and training of boys and girls, and though we agree with him as to the many defects in our present system, yet, it is only the out-cropping of our system of civilization (?), and until parents will cease allowing their children to be men and women before they lose their bread-and-butter odor, or get fairly dry behind the ears, there seems to be no help for it. Christianity takes very little part in their education; and so long as ministers of the gospel are engaged in every other pursuit than preaching the truths of the Bible, and the precepts of the Saviour; and so long, too, as physicians, afraid to lose the almighty dollar, lack sufficient moral courage to enlighten and guide, just so long must these evils last and increase. Yet despite all these drawbacks, the author's sweeping assertions and wholesale denunciations of boarding schools are exaggerated and unjust. The vast majority of boys and girls, whether they go to said places or not, acquire a certain amount of wickedness, and many are guilty of self-abuse, and continue it more or less until the development of their reasoning faculties teaches them its immorality, and the future danger and disgrace accruing from its continuance; but we believe that very few retain the habit after reaching mature life, and the percentage becomes still smaller after marriage. That he can point out the onanists of any school, is absurd, for the persons guilty of such an excess as to exhibit unequivocal objective signs are truly *raræ aves*, and all of those fearful evidences of mental wreck and physical decay so feelingly depicted, are seldom encountered by those who make special study of diseases of the sexual system. Every case he quotes, and every one he introduces from his own case book (one among many hundreds, he says,) are very extreme; and out of a large practice of seventeen years in hospitals, etc., under, and in company with the most learned men, and with a special attention given to such branches, we have seen but three or four examples, and none so extreme as our author's. We therefore dismiss this part of the subject, having given it more attention than it deserves.

And now as to "conjugal onanism" we desire to say a few words. He appears to mean by this term the incomplete performance of the marital act, no matter by what means accomplished, and very properly censures any such short-comings, but the grounds upon which he reprehends the perpetrators, are the physical injury to both parties; the diseases which it engenders; the unsatisfactory state of the woman after being stirred up, etc., etc. That it is a very unsatisfactory proceeding to both sides there is no question; and that "withdrawals" should be at a discount, is equally granted; but that cancer of the womb is generally occasioned by its congestion, not being relieved or washed out by the specific influence of the spermatic fluid, and that so many inflammatory conditions, not only of the womb, but its associated organs, should be occasioned likewise, the facts of physiology, and the etiology of the various pathological lesions, absolutely deny. It is now generally conceded, that excesses in sexual enjoyments are creative of the greater amount of inflammatory disorders, as much from the actual physical contact as from the nervous excitation. And that abortions are produced by the same causes, is also true; but that incomplete acts are more injurious than complete ones, we no more believe than that universal masturbation produces such terrible sequences among the community, or is carried to such frightful lengths, otherwise our hospitals, asylums and mad-houses would have insufficient accommodations for their numerous victims.

His doctrine of acquired resemblances between parents, he claims to be original, and holds these resemblances to be occasioned by the impregnation of the mother's blood at each successive pregnancy, by the father's, through the fœtus. This opinion is rather far-fetched and transcendental, for we had supposed that the fœtal blood was elaborated by the blood-making organs of the child itself, and simply received its purification from the maternal placenta; at the same time might transmit and receive, through mysterious nervous influences, some of those qualities and susceptibilities which it had obtained from the impregnating fluid. People who live together in perfect accord, and whose thoughts and desires often run in similar channels—who, in fact, get in perfect *rapport* with one another, very often acquire resemblances, not only physically, but mentally, even if not married; and this will in all probability account for these changes as plausibly as the blood-impregnation theory. The remainder of the work is well compiled, contains many good suggestions, and relieves in a measure the filthiness and absurdity of its foregoing portion; but, at the same time, we cannot help protesting that such literature should be published and circulated among the community. It is certain that the young man or woman that reads this work, will not only discredit much that is advanced, for their limited knowledge tells them differently, but it will excite a prurient curiosity to know more, and soon books of a still viler stamp will be devoured to their utter ruin and destruction.

For some time while reading, we were at an utter loss to discover to what particular sect or branch of medicine our author belonged; but the ear-marks of his vocation soon became apparent as we proceeded. And when we read through the case of the "Conjugal Hero," and the foot note explaining the philosophy of his wife's disease, we knew no physician educated in scientific medicine could have so jumbled up his pathology, and simply placed so many lesions of such important organs, and so many of them too, to the account of general or systemic irritation.

Besides, he went back on his own, by modestly disclaiming any cure in the case. When men for a succession of years treat symptoms as so many separate entities, they soon forget their pathological knowledge, if they ever had any, for there is not the least use for it; and hence are rarely honest enough to dis-acknowledge a cure in every case, even where diseases are self-limited, and get well by the *vis recuperandi naturæ*.

The dedication of this work is rather amusing, and that our readers may have the idea, we give it *in toto*, with some little change of our own:

To
Hon. William Sprague,
Ex-Governor of, and United States Senator
From, Rhode Island,
This book is dedicated.
Not to the Statesman (Ha! Ha!), the Politician (Ho, Ho!),
Nor the Millionaire ([?] Bosh!)
Nor even to the friend of former years;
But
To the Man (Bah!)
Who does prefer Gin and Sugar to Popularity.

——— Un dessein si'funeste,
S'el n'est pos digne d' Atree, cst digne de Thy este.
(*Crebillon.*)

